# Hæmoptysis

**Published:** 1877-09

**Authors:** John L. Cleveland


HÆMOPTYSIS.—*By John L. Cleveland, M.D.*—An individual has pulmonary hemorrhage; the physician is sent for, and in a vast majority of cases, the diagnosis is immediately made out—

phthisis. This idea prevails among the laity, propagated unquestionably by the professional opinion that exists on this point, to such an extent that with them consumption and pulmonary hemorrhage are nearly or quite synonymous; at least, hemorrhage is a sure forerunner of consumption. That this idea is erroneous, there is no doubt, and the physician who makes out a diagnosis of phthisis on this symptom alone, will find himself in error very frequently, perhaps in nearly one-half of his cases.

It is not our design to underestimate the importance of pulmonary hemorrhage or its gravity; we could scarcely do this if we desired, when we have before us the picture of terror and prostration that the hemorrhagic patient usually presents. It is only intended in this paper to treat of hæmoptysis of pulmonary origin. In the majority of cases, these hemorrhages are of bronchial origin, occurring in the smaller and terminal bronchi. In small hemorrhages the blood corpuscles pass through the uninjured capillary walls (diapedesis); large ones by rupture of vessels. Bronchial hemorrhages are not often of traumatic origin. They are caused for the most part by those agents which induce hyperæmia of the mucous membrane, whether the condition be due to increased flow of blood or to passive congestion.

We find them in acute bronchial catarrh accompanied with violent coughing, in whooping-cough, acute pneumonia, scarlet fever, measles, and small-pox. They are produced by the inhalation of irritating gases, by extremes of heat and cold, overexertion of the respirary organs by shouting, or speaking, or singing, or unusual bodily exertion; also, by deposits of tubercle in the mucous membrane, or parenchyma, of the lungs, or by heart disease, especially mitral insufficiency or obstruction. Bronchial hemorrhages, said to result vicariously, belong to this class produced by hyperæmia of the mucous membrane—those that are said to follow the cessation of the menses, or of hemorrhoidal bleeding. The latter, says Hertz, may be certainly ignored, for they exist only in the imagination of certain physicians. He thinks also that the influence of menstrual suppression on this form of hemorrhage should be accepted with a great deal of caution. Trousseau, in his lectures, says there is a class of cases in which the hæmoptysis is the result of what he calls "hemorrhagic deviation." He speaks of women subject to nervous attacks, who spit blood sometimes in considerable quantities, though they are suffering from derangement of the menstrual functions. Attentive examination reveals no disease of the respiratory or circulatory apparatus; the patients do not present any symptoms of cardiac or pulmonary disease, and when they reach the change of life, hæmoptysis ceases, never to return. Some women during pregnancy, and others during the whole time they are nursing, spit blood, the hemorrhage ceasing spontaneously after delivery, or at the end of lactation, as the case may be; nor is it symptomatic of pulmonary tubercle or cardiac disease. Trousseau also speaks of hæmoptysis being physiological to a certain extent, when it takes the place of a natural or accidental sanguineous discharge, which, for some cause, has been prevented from finding an exit by the usual outlets. Thus, in women in whom menstruation is suppressed or irregular, hæmoptysis is one of the most common forms in which hemorrhage occurs as a supplement of the menstrual flux. This, of course, includes hemorrhage supplemental to hemorrhoidal bleedings, which, Hertz says, have no existence except in the imaginations of some physicians. As to the existence of pulmonary hemorrhage as supplementary to menstruation, I am not prepared to deny its existence, but I must say I am skeptical in regard to it. I have never seen a case, though I have frequently heard them affirmed. I am quite convinced that in most of the cases of so-called supplementary hemorrhage, if they could be fully examined into, pathological conditions of the lungs or heart would be found sufficient to account for it. The mere circumstance should call for a close examination. Some of the following conditions would perhaps exist, though the existence of some would be difficult or impossible to prove without a post-mortem examination: Great fragility of the walls of the vessels; tubercles, or the primary elements of phthisis, or certain changes of the lung tissue, as softness and looseness of the parenchyma in consequence of chronic inflammatory conditions; or ulcerative or sloughing processes associated with the cavities of phthisis or bronchiectasis, or gangrene abscesses, or cancer of the lungs, or rupture of small aneurisms in cavities.

Then we have hemorrhagic infarction—a hemorrhage of the pulmonary tissue, confined within narrow limits, and which causes no destruction of the lung tissue. It frequently occurs with pulmonary emphysema and heart disease. "In fifty-nine cases of infarction, Bochdalek found organic disease of the heart in thirty-eight cases." The diagnosis of infarction is generally attended with some difficulty—in many cases it is impossible to arrive at a positive conclusion; and very frequently hemorrhagic infarctions are found in post-mortem examinations where they were not at all suspected. Infarctions of large size accompanied by well-marked symptoms are easily made out. In heart disease, for instance, with a sudden dyspnœa which may threaten suffocation, followed by a cough, with a sputa tinged with blood, with symptoms of circumscribed condensation of the lung, followed sometimes by pneumonia or pleurisy; in such cases we would readily make out infarction. The sputa from infarctions bear a certain resemblance to pneumonic sputa, but they are less tough, are almost always darker, and the expectoration continues much longer than in pneumonia. Finally, we have pulmonary apoplexy, so-called, or more properly, pulmonary rupture, in which the hemorrhage is so rapidly fatal that the physician seldom sees the patient before death, and when he does he is impotent as to any relief. The blood gushes forth in such quantities as sometimes to suffocate suddenly. The collapse is often so profound that the patient dies without coughing up any blood.

Pulmonary hemorrhage, while it may occur at any period of life, is more especially a disease of youth and early manhood. "Since the causes of hemorrhage belong unequally to various ages, the old opinion can rightly be maintained—the head in children, the thorax in youth, and the abdomen in later age most frequently furnish the causes for hemorrhage." (*Wagner.*) The rarity of hæmoptysis in children is worthy of remark. Huttenbrenner says, notwithstanding the destructive processes in the lungs of children, hemorrhage occurs very rarely. According to Vogel, when it occurs it is usually very profuse and is frequently fatal, bloody sputa seldom occurring. Hennock contradicts Vogel, and says bloody sputa are frequently found. Perhaps a reason for the rarity of pulmonary hemorrhage in children is the rarity of cavities in tuberculosis. Gerhardt states that in 265 cases of tuberculosis in children, 77 cavities were found.

Hæmoptysis, or true expectoration of blood, will frequently come on when the individual is apparently in perfect health, the patient being in repose; more frequently while undergoing exertion, without any premonition; sometimes there are prodromata—such as congestion of the head, headache, dizziness, palpitation, a feeling of oppression and thoracic fullness, increased frequency of the pulse, or sweetish, brackish taste in the mouth from spongy gums.

The effect of the hemorrhage upon the patient is usually out of all proportion to the loss of blood; even though the hemorrhage be slight, the patient will labor under great excitement and anxiety, the bravest giving way to terror and apprehension. The face becomes flushed, the pulse quickened, the expression alarmed, and if the hemorrhage is at all copious, prostration comes on, the face is covered with sweat, pulse grows weak, faintness and dimness of vision and ringing of the ears come on; usually they rapidly rally, unless the loss of blood has been very great. It may occur ar intervals, or one attack may be the end of it. For several days after the hemorrhage dark colored clots are raised, which gradually disappear. Every case of considerable hemorrhage that I have had an opportunity of observing has been followed by an increased temperature, varying from 99° to 102°, lasting from 7 to 14 days, usually being gradually reduced to the normal standard. This is in accordance with the opinion advanced by Niemeyer, that the blood in bronchial hemorrhage acts as an irritant to the lung; while in many or in most cases it is expectorated and absorbed, and the patient entirely recovers from any ill effects of the hemorrhage. In some cases, they do not rally, and perish in a few months from a phthisis florida—a galloping consumption; not from tuberculosis in the strict sense of the word, but from a form of consumption of which bronchial hemorrhage is the immediate cause. This is in accordance with the old view that classed hæmoptysis as one of the causes of consumption. This it appears might be true; the fact that hæmoptysis is a consequence of pulmonary disease does not invalidate it. Hertz and Ruehle, however, in Ziemssen, maintain to the contrary.

Relation between pulmonary hemorrhage and phthisis is one of coincidence rather than causation. It is absent in some of the most rapidly fatal cases, and is present in some that progress most slowly. The presence of hemorrhage is only a presumption in favor of phthisis, and it is always that except in rare cases. Niemeyer's views in regard to the relation between bronchial hemorrhage and pulmonary consumption, though not exactly in accordance with some of the more recent authorities, are certainly worthy of careful attention. They are summed up in the following paragraphs:

"Bronchial hemorrhage occurs oftener than is generally believed in persons who are not consumptive at the time of the bleeding, and may never become so.

"Copious bronchial hemorrhage frequently precedes consumption, there being, however, no relation of cause and effect between the hemorrhage and the pulmonary disease. Here both events spring from the same source—from a common predisposition on the part of the patient, both to consumption and bleeding.

"Bronchial bleeding may precede the development of consumption as its cause, the hemorrhage leading to chronic inflammation and destruction of the lung.

"Hemorrhage from the bronchi occurs in the course of established consumption more frequently than it precedes. It sometimes, though rarely, appears where the disease is yet latent.

"When the bronchial hemorrhage takes place during the course of consumption, it may accelerate the fatal issue of the disease, by causing chronic destructive inflammation."

Ruehle explains the fact of the rapidly fatal termination of some cases, that were apparently well up to the time of the hemorrhage, by the statement that large vomicæ sometimes exist, the patient at the same time imagining himself in perfect health, and is so regarded by others. A case of immediately fatal hemorrhage is narrated, where a cavity was found containing the ruptured vessel, and yet no previous symptoms of disease had been noticed. Prognosis of bronchial hemorrhage, so far as any immediate danger is concerned, is almost always favorable. The ultimate effects of hemorrhage will depend upon the quantity, frequency, and the existence of vomicæ.

When vomicæ do exist, hemorrhage always is attended by more or less danger, not so much from bleeding to death, though that does sometimes occur, as from the anæmia and prostration that follow, which may hasten a fatal result.

Profuse hemorrhages always suggest cavities, even though the subject may be in apparent perfect health. Ruehle thinks we have no reason to think that the hemorrhage comes from the bronchi, when it is copious, has never seen a case of this kind, and doubts their occurrence. The size of the cavities bear no direct relation to the copiousness of the hemorrage. Fortunately, hemorrhage is usually self-limiting, from contraction of the muscular coats of vessels; from coagulability; from diminished pressure; from loss of blood, or syncope, or both; from increased flow of lymph from the thoracic duct, due to the loss of blood, thereby increasing coagulability. Hemorrhage from infarctions, while it is of no consequence as hemorrhage, it indicates a condition of the lung that may result in serious inflammatory and degenerative processes; of couse, when the result of pyæmia or metastatic abscesses, it bodes evil. The hemorrhage of pulmonary appoplexy, of course, is always fatal.—*Clinic.*

